# B-Lymphocytes in the Pathophysiology of Pancreatic Adenocarcinoma

**DOI:** 10.3389/fimmu.2022.867902

**Published:** 2022-03-14

**Authors:** Claudia Minici, Sabrina Testoni, Emanuel Della-Torre

**Affiliations:** ^1^Università Vita-Salute San Raffaele, IRCCS San Raffaele Scientific Institute, Milan, Italy; ^2^Pancreato-Biliary Endoscopy and Endosonography Division, IRCCS San Raffaele Scientific Institute, Milan, Italy; ^3^Division of Pancreatic Surgery, Pancreas Translational and Clinical Research Center, IRCCS San Raffaele Scientific Institute, Milan, Italy; ^4^Unit of Immunology, Rheumatology, Allergy and Rare Diseases (UnIRAR), IRCCS San Raffaele Scientific Institute, Milan, Italy

**Keywords:** B lymphocyte, pancreatic adenocarcinoma, PDAC - pancreatic ductal adenocarcinoma, B cells, fibrosis, cancer associated fibroblast (CAF)

## Abstract

Pancreatic adenocarcinoma is highly infiltrated by B lymphocytes but the relevance of these immune cells in tumor development has been surprisingly overlooked until recently. Based on available evidence from other solid tumors, interaction between B lymphocytes and neoplastic cells is probably not uniformly stimulatory or inhibitory. Although presentation of tumor antigens to T cells and production of antitumor immunoglobulins might intuitively suggest a prominent tumor suppressive activity, specific subsets of B lymphocytes can secrete growth factors for neoplastic cells and immunosuppressive cytokines thus promoting escape from immunosurveillance and cancer progression. Because many of these mechanisms might also be implicated in the development of PDAC, and immune-modulation of B-cell activity is nowadays possible at different levels, determining the role of B-lymphocytes in this lethal cancer becomes of utmost importance to design novel therapeutic strategies. This review aims to discuss the emerging role of B cells in PDAC tumorigenesis, progression, and associated stromal reaction.

## Introduction

Pancreatic ductal adenocarcinoma (PDAC) is a highly aggressive malignancy with a 5-year survival rate of 5-7% and represents the third leading cause of cancer related mortality (1, 2). Pancreatic ductal adenocarcinoma mainly arises from two types of precursor lesions, pancreatic intraepithelial neoplasia (PanIN) and intraductal papillary mucinous neoplasms (IPMNs) that eventually transform into invasive carcinoma after progression from low to high-grade dysplasia (3, 4). Aside from palliative care or surgery in eligible patients, current treatment options are based on DNA-damaging agents but still offer a life expectancy of less than 40% at three years (5). Failure of available chemotherapy regimens has been traditionally attributed to mechanisms of chemoresistance intrinsic to neoplastic cells and to the characteristic stromal reaction that hampers effective drug delivery into the tumor (6–9).

The immune system plays an equally important role in modulating PDAC progression with opposing influence of tumor promoting and tumor suppressing immune cells (6–9). Specifically, while CD8^+^ T cells, Th1-type CD4^+^ T cells, and natural killer (NK) cells bear antitumor activity, regulatory T (Treg) cells, Th2-type CD4^+^ T cells, myeloid-derived suppressor cells (MDSCs), and tumor-associated macrophages (TAMs) have been shown to create a highly immunosuppressive microenvironment that decreases PDAC immunogenicity (7–16). Indeed, cytotoxic T and NK cells infiltrate premalignant IPMN lesions from early stages, but they progressively decrease in number and antitumor activity in parallel with the accumulation of immunosuppressive elements (17).

Pancreatic adenocarcinoma is also highly infiltrated by B lymphocytes but, in contrast to other malignancies, the relevance of these immune cells in tumor development has been surprisingly overlooked until recently. Evidence of B-cells involvement in the progression of solid tumors, in fact, has been appreciated as early as the late 1970s and subsequently explored in a number of non-pancreatic cancers (18–23). In breast, ovarian, and gastric adenocarcinoma, for instance, a prominent B lymphocytic infiltrate correlates with better outcomes (24, 25). Similarly, in non-small cell lung cancer, the presence of tertiary lymphoid structures (TLS) enriched in B cells predicts longer patient survival (26). Yet, B-cell deficiency or depletion reduced disease burden in mouse models of sarcoma, colorectal cancer, melanoma, and squamous carcinomas, suggesting equally relevant tumor promoting properties of the B-cell compartment (27–33). Together, these data support a complex and nuanced interaction between B lymphocytes and neoplastic cells that is probably not uniformly stimulatory or inhibitory across a wide range of cancers. Although presentation of tumor antigens to T cells and production of antitumor immunoglobulins might intuitively suggest a prominent tumor suppressive activity, specific subsets of B lymphocytes can secrete growth factors for neoplastic cells and immunosuppressive cytokines thus promoting escape from immunosurveillance and cancer progression (34–40). The ultimate tumor promoting or tumor suppressing effect of B cells might also be influenced by a number of variables related to the experimental setting, including the B-cell depletion strategy adopted, the mouse model used, and whether the study is focused on peripheral or on tumor-infiltrating B lymphocytes (TIL-B).

Because many of these mechanisms might also be implicated in the development of PDAC, and immune-modulation of B-cell activity is nowadays possible at different levels with a portfolio of targeted treatments, determining the role of B-lymphocytes in this lethal cancer becomes of utmost importance to design novel therapeutic strategies. In the present review we discuss the emerging role of B cells in PDAC tumorigenesis, progression, and stromal reaction. In addition we examine the mechanisms by which B cells might promote as well as inhibit antitumor immunity. To place our work in context, an overview of the available mouse models for studying B cells in PDAC will also be provided as well as hints from targeted B-cell approaches under evaluation in clinical trials.

## Mouse Models for Studying B-Lymphocytes in Pancreatic Adenocarcinoma

There are several different genetically modified mouse tumors and xenograft preclinical model systems available to study pancreatic cancer from its pre-neoplastic stage to its metastatic form (41–48). These models use a variety of approaches to target the expression of mutant or endogenous specific genes and, as a result, they develop a broad spectrum of pathologic changes.

In general, as mutations in Kras have been reported in 90% of human PDAC and in more than 95% of PanIN lesions (49–51), most mouse models are based on the alteration of this oncogene. Kras mutations alone are, however, not sufficient to induce PDAC progression to the invasive stage, and different tumor suppressor genes are typically deleted or inactivated to generate metastatic disease including Ink4, p53, Smad4 or TGFβ (52–56). Of note, all these different types of murine models are immunologically intact thus allowing concomitant characterization of the immune response during PDAC progression. Yet, data regarding the composition and functional status of the B-cell infiltrate are available only for KPC, KC mice, and orthotopic models.

The KPC mouse (KrasG12D/+;Trp53R172H/+;Pdx-1-Cre) represents the gold-standard of PDAC preclinical models and is generated by placing a Cre recombinase G12D Kras mutated gene under the control of the pancreas-specific promoter Pdx1 together with silencing of the tumor suppressor p53 gene (57). KPC mice have a median survival of 5 months and faithfully recapitulates human disease in terms of spontaneous progression through PanIN, desmoplastic stromal reaction, and metastatic spread (57). In addition, because Kras expression directly induces Treg cells differentiation, the KPC mouse also offers the opportunity to study the progressive impairment of anti-tumor immune responses that occur in the human counterpart (58, 59). The pancreas of the KPC mouse is strongly infiltrated by B lymphocytes and the proportion of B-cells observed in KPC tumors is comparable to that reported in PDAC patients (60). Of note, B lymphocytes are found not only enmeshed within stromal elements but also clustered in germinal centers-like aggregates, suggesting that the KPC model elicits antigen dependent immune-reactions at disease site (60). Indeed, in analogy to human PDAC, most B-cells infiltrating KPC tumor are memory B-cells, show an activated phenotype, and produce a significant amount of immunoglobulins (60). Lower numbers of regulatory B-cells (Breg) and plasma cells are also observed. Given the analogies with human disease and the capacity to elicit an organized local inflammatory response, the KPC mouse currently represents the best available model to physiologically address the relationship between the B-cell compartment and the various cellular and stromal components of PDAC.

Another related model, the KC mouse (KrasG12D/+;Pdx-1-Cre), expresses only mutant Kras and consequently develops PDAC at a significantly slower rate (61). Mice are histologically normal up to 5 months and do not develop PanIN lesions until after 8 weeks (52). In KC mouse, B cells only modestly infiltrate pancreatic tissue, distribute in proximity to PanIN, and prominently exhibit a B1 and B2 mature phenotype on flow-cytometry. KrasG12D pancreata show diminished frequencies of B2 cells and increased frequencies of B1 cells compared to wild type mice, and their number decreases during carcinogenesis (60, 62). In addition, B1 cells show no to low memory responses, express IgM in greater quantities than IgG, and their B-cell receptors are polyspecific, suggesting a lower immunogenicity of KC lesions compared to KPC mice (60, 63). Because of the latency of tumor formation and the paucity of B-lymphocytic infiltrate, the KC mouse is considered suitable to characterize B-cell contribution to PDAC development rather than PDAC progression. The simultaneous Kras activation and p48/Hif1a deletion (p48-Cre;LSL-KrasG12D;Hif1afl/fl) in the pancreatic epithelium, for instance, has been recently exploited to address B-cell implication in pre-neoplastic PanIN lesions (64). In this model both the percentage and absolute number of B cells were dramatically increased compared with KC and wild type mouse, and, as opposed to the KC model, they did not decrease during carcinogenesis (64). In addition, compared to normal mice, the proportion of CD19hiCD1dhiCD5+ Breg cells was higher both in KrasG12D and KrasG12D;Hif1aKO pancreata, with no significant differences between the two models (64).

A third model to study B-cells in PDAC is the orthotopic model, which is generated by the injection of syngeneic tumor cells derived from primary pancreatic carcinomas of transgenic mice into the pancreas of wild type mice, typically C57BL/6 mice. Orthotopic tumors form rapidly in approximately one month, contain more tumor cells than stromal elements and exhibit significant infiltration by CD45+ leukocytes compared with wild-type mice (52). Yet, due to accelerated disease progression, B-cell infiltration occurs to a far lesser extent and with delayed kinetics than in KPC tumors both in density and proportion, and germinal center (GC)-like structures are typically not observed (60). This evidence suggests that the immunological stimulus provided by tumor cell lines in the orthotopic PDAC model is also less proficient in inducing antibody-secreting plasma cells than KPC mice. Indeed, the activation profile of infiltrating B-cells is much reduced in this model compared to the KPC mouse, and the deposition of immunoglobulins is virtually absent (60). Accordingly, B-cells predominantly bear an IgM transitional and B1 phenotype, with IgM^low^ memory B cells, Breg cells, plasmablasts, and plasma cells observed to a lesser extent, not significantly different from healthy pancreas (60). Still, although the faster tumor kinetics compared to KPC models probably prevent full B-cell activation, orthotopic tumors seem equally capable of shaping the phenotype of infiltrating B cells as pancreatic B lymphocytes possess a remarkably different activation profile than splenic B cells from the same tumor-bearing mice, including the expression of proinflammatory cytokines and chemokines involved in T-cell recruitment (60).

The relevance of B lymphocytes in orthotopic PDAC models has been recently assessed also by injecting syngeneic pancreatic cancer cell lines into B-cell deficient mice, either μMT or J_H_T mice (65, 66). The μMT mouse is homozygous for the *Ighm^tm1Cgn^* targeted mutation and has no expression of membrane-bound IgM. Similarly, the J_H_T mouse is homozygous for the *Igh-J^tm1Cgn^* targeted mutation that causes a deletion in the J segment of the Ig heavy-chain locus and prevents the expression of IgM or IgG. The results of both mutations are mouse models that have no mature functional B cells in the bone marrow or periphery due to blocked B-cell differentiation at the precursor stage. These models have both advantages and shortcomings. On the one hand, in fact, orthotopic PDAC models in B-cell deficient mice allow selective evaluation of the contribution of specific B-cell subsets by injecting them intravenously or directly into the recipient pancreas. On the other hand, however, B-cell deficient mice bear intrinsic derangement not only of humoral immune responses but also of cellular immunity, making it possible that intrinsic characteristics of B-cell deficient mice may contribute to orthotopic tumor development, rather than the loss of B cells *per se* or the effect of a single B-cell subpopulation of interest (60). Finally, the orthotopic model could be also exploited to directly assess the contribution of human B lymphocytes to tumour growth. This model would theoretically imply xenografts of human PDAC cell lines in immunodeficient mice (typically, NOD-scid IL-2 receptor gamma^null^ mice) but we are not aware of studies using this model until the time of writing, and caveats related to the global impairment of the murine immune system remains to be considered. Still, although labor intensive, technically challenging, and expensive, orthotopic xenograft of PDAC represents a valid approach to recapitulate human disease *in vivo* and to increase the translational relevance of its findings.

## Spatial Distribution of B-Lymphocytes in Pancreatic Adenocarcinoma

Spatial compartmentalization of immune cells within the tumor tissue (whether peritumoral, intratumoral, diffuse, or organized in tertiary lymphoid structures) is considered an important determinant of cancer progression (67). In particular, tertiary lymphoid structures are considered crucial for the recruitment of tumor-infiltrating T lymphocytes (TILs), for B-T cells interaction in GCs, and for the activation of tumoricidal cytotoxic responses associated with better clinical outcomes (68–72). Germinal centers are essential also for the development of effective humoral responses whereby naïve B-cells are activated after antigen encountering, undergo affinity maturation under the guidance of follicular helper T (Tfh) cells, and differentiate in antibody producing plasmablasts/plasma cells (73, 74).

**Figure 1:**
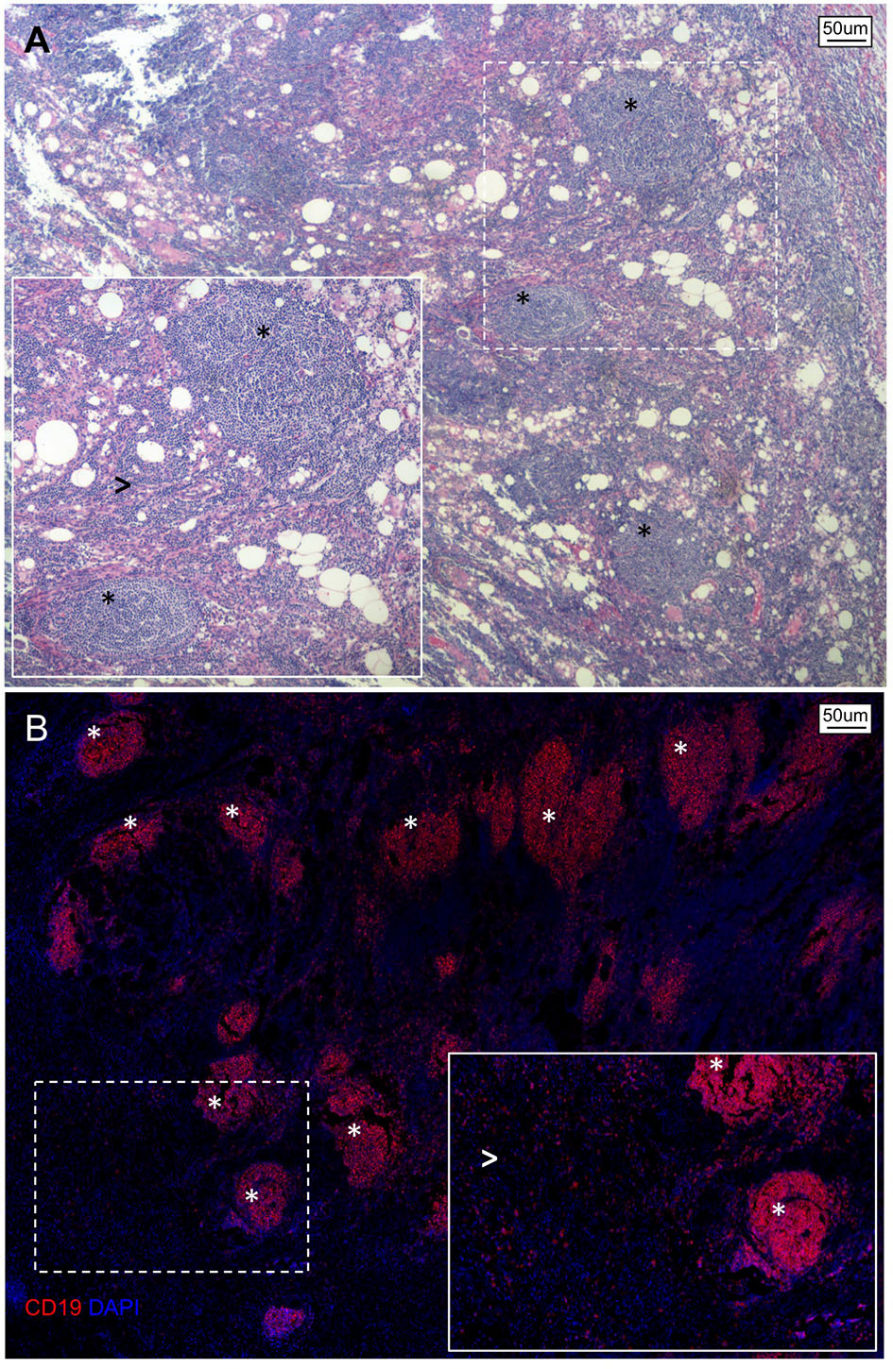
Spatial organization of B lymphocytes in pancreatic adenocarcinoma. **(A)** Hematoxylin/Eosin stain and **(B)** immunofluorescence staining of a representative tissue section of PDAC showing an abundant infiltrate of B lymphocytes organized as either multiple lymphoid structures (asterisks) or spread within the neoplastic lesion and tumor stroma (arrowheads).

In the context of PDAC, B lymphocytic infiltrate have been preferentially observed either scattered within the stromal microenvironment (TILs) or organized in ectopic lymph node-like TLS (75–78) (**Figure 1**). According to a retrospective analysis of 104 histological PDAC specimens, this bimodal spatial distribution was independently associated with patient survival, suggesting that compartmentalization of B-lymphocytes in the pancreatic tissue reflects opposite immunological commitments (75). Specifically, while TILs interspersed within the tumor stroma were associated with worse prognosis, the presence of intratumoral TLS correlated with longer overall and progression-free survival (75). Accordingly, PDAC specimens rich in TLS showed upregulation of genes related to GC reaction, B-cell activation, and proliferation including activation-induced cytidine deaminase (AICDA), Ki67, and CD27 (75). Conversely, PDAC specimens with prominent TILs infiltrate exhibited increased expression of immunosuppressive genes, including programmed cell death-1 ligand (PDL1) and transforming-growth factor-b (TGF-b) (75). PDAC specimens rich in TLS also showed increased expression of genes related to T-cell activation and proliferation (namely, CD8, IL-2, IL-7, and IL-12), and pancreatic infiltration of cytotoxic CD8^+^ T cells was observed in orthotopic PDAC models after B-cell depletion (75). Notably, GC reactions occurring in TLS^+^ PDAC specimens are enriched in class-switched memory B cells, effector memory CD4^+^ T-cells, and NK cells suggesting that durable immunological memory is associated with a longer survival (78). Similarly, GC reactions in TLS^+^ tumors express significantly more MHC class I-restricted neoantigens and somatic hypermutations in B-cells, indicating that a more specific humoral immune response also represents an important variable for patient outcome (78).

All together, these results confirm that B-cells bear prognostic relevance in human PDAC and suggest that their ability to influence tumor progression depends on whether they are organized in functionally proficient tertiary lymphoid structures with mature GC reactions (*tumor-suppressing*) or scattered at the tumor-stroma interface (*tumor promoting*). Of note, while distribution of B-lymphocytes in PDAC is likely orchestrated by chemotactic factors secreted by neoplastic and/or stromal cells, the dynamics of B-cells compartmentalization during tumor development remain to be fully characterized, and possibly implies antagonizing functions of coexisting TILs and TLS as well as a progressive loss of TLS along with increasing PDAC aggressiveness.

## B-Lymphocytes and the Tumorigenesis of Pancreatic Adenocarcinoma

Studies focused on T-cell responses in PDAC have shown that the progression of pre-malignant IPMN lesions to invasive cancer are associated with a consistent modification of the immune landscape whereby cytotoxic tumor-suppressing CD8^+^ T-cells, Th1 cells, and NK cells are progressively supplanted by immunosuppressive and tumor-promoting Th2 cells and Treg cells during the invasive stage of pancreatic cancer (17, 79).

The role of B-lymphocytes in early stages of PDAC development has been evaluated in few orthotopic mouse models of pancreatic ductal epithelial cells expressing oncogenic Kras (KrasG12D-PDEC) KPC derived cell lines injected into the pancreas of μMT or J_H_T mice and in organoids (65, 66, 80, 81). Injection of KrasG12D-PDEC or KPC derived cell lines into wild-type pancreas, in fact, typically leads to the accumulation of B-lymphocytes in close proximity to evolving tumor lesions, probably as a consequence of the secretion of the B-cell chemotactic molecule CXCL13 by stromal cells in the context of the growing pancreatic neoplasia (66). Conversely, orthotopic implantation of either KrasG12D-PDEC or KPC derived cell lines in B-cell deficient mice was associated with a significant reduction of tumor lesions compared to wild type mice, and adoptive transfer of B-cells from littermate controls into μMT animals prior to orthotopic injection of neoplastic cells rescued tumor growth together with *de novo* infiltration of transferred B-cells (66, 80). These findings suggest that the epithelium “in transformation” instructs the recruitment of B-cells and support an essential role of B-lymphocytes in establishing a pro-tumorigenic microenvironment. Accordingly, prevention of early B-cells recruitment in KrasG12D-PDEC orthotopically implanted mice with anti-CXCL13 treatment also resulted in reduced growth of the orthotopic lesions reinforcing possible tumor promoting properties of B-lymphocytes in the initial stages of PDAC development (66). In another study of primary human pancreatic tumors and KC mouse model crossed to Hif1α^fl/fl^ the hypoxia inducible factor-1α (HIF1α) was found to be highly expressed during the pre-invasive stage of PDAC and deletion of HIF1α accelerated PDAC development (61). Elimination of HIF1α resulted in an increased secretion of the B-cell chemoattractants CXCL13 and CCL21 and in intratumoral accumulation of CD19^+^ B-lymphocytes during early pancreatic neoplasia. On the other hand, depletion of B-cells reduced the progression of PanIN lesions and the development of invasive carcinomas in tumor-bearing mice further supporting the involvement of human B-lymphocytes in PDAC tumorigenesis. Yet, besides this correlative evidence, the exact mechanisms driving B-cell mediated promotion of early tumor development and progression remain elusive.

## B-Lymphocytes and the Humoral Response in Pancreatic Adenocarcinoma

Besides functioning primarily as antigen presenting cells and providers of surviving signals to T-cells, B-lymphocytes may contribute to PDAC associated immune response by producing autoantibodies against immunogenic tumor-associated antigens (TAAs). Tumor-associated antigens are proteins expressed by tumor cells capable of activating cellular and humoral immune responses *via* several mechanisms including, among others, mutations resulting in the formation of neoantigens (82). Pancreatic cancer is, however, a poorly immunogenic tumor due to a low mutational burden and antigenic targets relevant for PDAC progression are currently poorly characterized (83–85).

Yet, in the attempt to identify potentially useful biomarkers, several autoantibodies have been described in patients’ sera (86–89) (**Table 1**). In a study screening serum IgG against proteins from pancreatic cancer cell lines (CF-PAC-1, MiaPaCa-2, and BxPC-3), two-dimensional gel electrophoresis followed by mass spectrometry identified a number of metabolic enzymes and cytoskeletal proteins as the main target of autoantibodies in PDAC patients (89). Most of these TAAs such as triosephosphateisomerase 1 (TPIS), glucose-6-phosphate-dehydrogenase (G6PD), isocitrate-dehydrogenase (IDHC), keratin 10 (K1C10), cofilin-1 (COF1) and transgelin (TAGL) were also up-regulated in PDAC tissue suggesting an attempt of the immune system to target molecules involved in tumor metabolism and metastasis (89). Additional auto-antigens identified by different proteomic and array approaches include mucin 1 (MUC1), Rad51, insulin, proteins expressed in b-islet cells, phosphoglycerate kinase 1 (PGK1), DEAD-box protein 48 (DDX48), and the tumor suppressor p53, with MUC1, PGK1, and DDX48 having shown some degree of utility for the differential diagnosis of PDAC with chronic pancreatic disorders (86–88, 90, 91). Autoantibodies against PDAC-associated antigens have been, however, observed in up to only 30% of cases and, although their presence suggests that targeted proteins may play a role in carcinogenesis, none has gained sufficient evidence to be exploited for immunotherapeutic purposes (85–89, 91).

**Table 1:** Tumour associated antigens in pancreatic adenocarcinoma.

Antigen	PDAC patients (%)	Function	Experimental details		Ref
			Antigen source	Identification technique	
﻿Phosphoglycerate kinase 1 (PGK1)	20.8%	Up-regulated by HIF-1α in response to hypoxia to provide energy for tumor cell proliferation Suppressor of proangiogenic factor such as VEGF Triggers tumor metastasis by increasing expression of β-catenin, CXCR4 and CXCL12	PANC1 cells	2-D liquid separation/microarray LC-MS/MS	(85)
﻿DEAD-box protein 48 (DDX48)	63.6%	Member of the DEAD-BOX family with RNA helicase activity Recruited to the exon-junction complex during splicing	HEp-2 cells	Oone-dimensional Western blot 2-D PAGE and LC-MS/MS	(86)
p53	18.2%	Increased expression after DNA damage to induce growth arrest, DNA repair and apoptosis	–	ELISA	(87)
Calreticulin isoforms	58%	Ca2+-binding proteins found in the endoplasmic reticulum (ER) lumen Two major functions: chaperoning and regulation of Ca(2+) homoeostasis	PANC1 cells	2-D PAGE/Western blot LC-MS/MS	(88)
﻿Mucin 1 (MUC1)	Unknown	Transmembrane glycoprotein involved in cell–cell and cell–extracellular matrix interactions Circulating anti-MUC1 IgG antibodies could be a favorable prognostic factor in PDAC patients	–	ELISA ﻿	(89, 90)
Rad51	7%	Recombination and repair of DNA double strand breaks	﻿Rad51 over-expressing cell lines	Western blot	(91)
b-islet cells	57%	Detectable serum titres of both the autoantibodies in PDAC patients correlate with worse outcome	–	ELISA	(91)
Insulin	48%				
Triosephosphate isomerase 1 (TPIS)	14-23%	Converts dihydroxyacetone phosphate into glyceraldehyde 3-phosphate in the glycolytic pathway Up-regulated by the enhanced glycolytic activity of tumour cells Favours high levels of pyruvate and lactate production	﻿CF-PAC-1, MiaPaCa-2, BxPC-3 cells	2-D PAGE/Western blot	(88)
Retinal dehydrogenase 1 (AL1A1)	20%	Key role in the biosynthesis of retinoic acid, which in turn acts in cell signalling pathways	﻿CF-PAC-1, MiaPaCa-2, BxPC-3 cells	2-D PAGE/Western blot	(88)
Glucose-6-phosphate 1-dehydrogenase (G6PD)	13%	Enzyme of the pentose phosphate pathway Catalyses the conversion of glucose-6-phosphate to gluconolactone 6-phosphate Overexpressed in several types of cancers and correlate with worse prognosis	﻿CF-PAC-1, MiaPaCa-2, BxPC-3 cells	2-D PAGE/Western blot	(88)
Elongation Factor Tu (EFTU)	11%	G-protein that catalyses the binding of aminoacyl-tRNA to the A-site of the ribosome	﻿CF-PAC-1, MiaPaCa-2, BxPC-3 cells	2-D PAGE/Western blot	(88)
Isocitrate dehydrogenase (IDHC)	13%	Enzyme of the citric acid cycle to create molecules that are used for cellular energy Catalyses the oxidative decarboxylation of isocitrate to 2-ketoglutarate to generate NADPH from NADP+	﻿CF-PAC-1, MiaPaCa-2, BxPC-3 cells	2-D PAGE/Western blot	(88)
Keratin 10 (K1C10)	21%	Member of the Keratins family, fibrous proteins that form the structural framework of cells Partner of keratin 1 to form keratin intermediate filaments in all epithelial cells	﻿CF-PAC-1, MiaPaCa-2, BxPC-3 cells	2-D PAGE/Western blot	(88)
﻿Transgelin (TAGL)	27%	Member of the calponin family induced by transforming growth factor beta Actin-crosslinking protein, earliest markers of differentiated smooth muscle cells Elevated levels correlate with aggressive tumour behaviour, advanced stage, and poor prognosis	﻿CF-PAC-1, MiaPaCa-2, BxPC-3 cells	2-D PAGE/Western blot	(88)
Cofilin-1 (COF1)	27%	Actin depolymerizing protein involved in various functions including cytokinesis, endocytosis, apoptosis and cell migration Essential for cell directionality and metastasis of various solid tumours in response to chemotactic or growth-factor stimulation	﻿CF-PAC-1, MiaPaCa-2, BxPC-3 cells	2-D PAGE/Western blot	(88)

Over and beyond these important aspects pertaining antigen specificity of circulating autoantibodies, humoral response in PDAC patients bears additional features related to IgG subclasses, isotype, and glycosylation profile of secreted immunoglobulins that have relevant prognostic implications. The presence of IgG1^+^ memory B-cells in TLS^+^ PDAC specimens, for instance, associates with longer overall survival, while a prominent infiltration of intratumoral IgG4^+^ plasma cells positively correlates with poor histological grade and worse overall survival, supporting the importance of IgG class-switching for effective anti-tumor humoral immune responses (78, 92). Among the four human IgG subclasses, in fact, IgG1 shows the highest proficiency at triggering immune system components through complement activation, binding of Fc receptors on the surface of effector cells, and engagement of antibody-dependent cell-mediated cytotoxicity (ADCC) (93). On the other hand, IgG4 is considered an “anti-inflammatory” subclass due to its poor ability to fix complement and to activate effector cells (93). Of note, IgG4 antibodies are typically produced in the context of IL-10 and IL-4 driven “modified” Th2 immune responses such as those observed in the microenvironment of PDAC and of other solid tumors (94). Class switching from IgG1 to IgG4 may, therefore, represent an additional immune escape mechanism by which PDAC shapes humoral immunity during its progression to restrain anti-tumor immune responses. Indeed, although overlooked in the setting of pancreatic cancer, this hypothesis has been verified in melanoma patients whereby IgG4 antibodies significantly impaired the potency of tumoricidal IgG1 *in vivo* in a xenograft mouse model (95). IgE are also immunoglobulins classically associated with Th2 immune responses but, as opposed to IgG4, they have been shown to participate to tumor cells killing in ovarian cancer *via* ADCC, and their serum concentration correlates with longer overall survival in glioblastoma patients (96, 97). In PDAC patients, IgE levels are significantly increased compared to healthy controls, recognize self antigens, and bear *in vitro* ADCC activity against pancreatic cancer cells lines but their prognostic relevance has never been assessed in mouse models (98). Indeed, ADCC in PDAC appears markedly attenuated by tumor-derived exosomes that harbor B-cell targets, bind circulating autoantibodies, and exert decoy function against complement-mediated cytotoxicity (99–102).

Finally, as changes in IgG glycoforms have been reported during the progression and metastasis of solid tumors, glycosilation profile of IgG subclasses in PDAC patients has also been evaluated (103–105). Glycosylation is, indeed, one of the most relevant post-translational modifications of human immunoglobulins, and modification of the N-glycan structure in the Fc region of IgG significantly profoundly affect their functions (106). Increased galactosylation and sialylation, for instance, are known to exert anti-inflammatory properties while reduced fucosylation enhances ADCC activity (106). Compared with healthy controls PDAC patients exhibit a significantly increased IgG agalactosylation and a significantly decreased IgG fucosylation and sialylation (103–105). In addition, compared with early stage, advanced PDAC shows reduced galactosylation and increased fucosylation of serum IgG1 confirming the association between IgG glycosylation and tumor progression that has been previously described in gastric and prostate cancers (103–105).

All together, this evidence indicates that PDAC is capable of orienting the anti-tumor humoral response but further studies are needed to unveil the role of different glycoforms and IgG subclasses in cancer progression, possibly in relation with antigen specificity of secreted autoantibodies.

## B-Lymphocytes and the Tumor Stroma in Pancreatic Adenocarcinoma

In apparent contrast with PDAC aggressiveness, the majority of the tumor volume is not made of malignant cells, but of a desmoplastic reaction consisting of cancer-associated fibroblasts (CAFs), extracellular matrix (ECM), and immune cells collectively termed as “tumor stroma” (107). Tumor stroma is commonly believed to support PDAC growth by promoting a hypovascular hypoxic microenvironment, escape from immune-surveillance, chemoresistance, and metastatic dissemination (1, 108, 109). Although B-cells represent a salient feature of PDAC microenvironment, their interaction with other stromal elements has not been evaluated until recently.

In a study exploiting co-cultures of B-cells and CAFs from PDAC patients to assess the contribution of B-lymphocytes to the characteristic stromal desmoplasia, unexpected pro-fibrotic properties emerged from functional and transcriptomic analyses (110). In particular, B-lymphocytes purified from PDAC patients (i) induced collagen production by CAFs *via* soluble factors (ii) up-regulated genes associated with epithelial-to-mesenchymal transition in co-cultured CAFs, and (iii) produced the pro-fibrotic molecule platelet derived growth factor-B (PDGF-B) (110). Circulating plasmablasts were shown to be the most proficient B-cell subset at stimulating collagen production by CAFs, as opposed to naïve B-cells and memory B-cells, and expressed a set of genes implicated in the “activation of fibroblast proliferation” including the collagen genes COL1A1 and COL1A2, insulin-like growth factor-1 (IGF-1), and lysyl oxidase homolog 2 (LOXL2) (110, 111). Of note, plasmablasts are CD19^+^CD20^−^CD27^+^CD38^+^ cells arising from GCs after affinity maturation from CD20^+^ naive precursors and differentiate into antibody secreting long-lived plasma cells after homing to inflammatory niches or to the bone marrow (112). They circulate for prolonged periods in the setting of chronic antigen exposure but are infrequently observed in the peripheral blood of healthy individuals (113, 114). Circulating plasmablasts were significantly expanded in the peripheral blood of treatment-naïve PDAC patients compared to healthy individuals and LOXL2 expressing plasmablasts were shown to infiltrate pancreatic lesions (110). LOXL2, on the other hand, is an enzyme that catalyzes the cross-linking of collagen components in the ECM, thus contributing to the overall tensile strength of collagen bundles and, indirectly, to the differentiation of quiescent fibroblasts into activated myofibroblasts through activation of mechanoceptors (111, 115, 116). LOXL2 has been also implicated in carcinogenesis, resistance to gemcitabine, and invasiveness of pancreatic cancer cells, and its inhibition has been shown to reduce tumor volume and metastases in preclinical studies (117).

All together these preliminary evidence demonstrates a direct interaction between B-lymphocytes and CAFs, and suggests an overlooked contribution of B-cells to PDAC pathophysiology *via* orchestration of stromal activation over and beyond their primary role in humoral response. Whether these pro-fibrotic properties are ultimately tumor promoting or tumor suppressing remains to be fully ascertained as recent *in vivo* experiences with selective knock out of ECM components in KPC mouse models have raised the possibility that stromal elements does not uniformly promote or restrain PDAC growth, increasing the degree of complexity around tumor-stroma crosstalk (118). Indeed, a slower tumor growth in B cell deficient JH^−/−^ mice orthotopically injected with syngeneic PDAC cell lines was paralleled by a significantly reduced desmoplastic reaction compared to littermate controls, indirectly supporting possible tumorigenic interactions between B-lymphocytes and pancreatic fibroblasts (65).

## Tumor Promoting Role of B-Lymphocytes in Pancreatic Adenocarcinoma

B-lymphocytes are generally considered tumor promoting immune cells based on several evidence from a number of solid cancers including prostate cancer (119, 120), liver cancer (121), and breast cancer (122) where they have been shown to sustain tumor growth and metastasis (38). In particular, the tumorigenic role of B-lymphocytes is largely attributed to Breg cells, a B-cell subset with primary immunosuppressive properties (123) that, in addition to Treg cells, hampers antitumor immune response.

Regulatory B-cells represent less than 10% of total circulating B-lymphocytes in healthy subjects and physiologically regulate immune homeostasis mainly, but not exclusively, through the production of the anti-inflammatory cytokine IL-10 (124). Human Breg cells typically express CD24, CD1d, and variable levels of CD27, but subsets with overlapping phenotypes and functions have been identified (125, 126). B-regulatory cells exert their immunosuppressive functions in different ways including (i) induction of CD4^+^CD25^+^FOXP3^+^ Treg cells (27, 38); (ii) inhibition of inflammatory cytokines release by macrophages and monocytes; (iii) inhibition of T_H_1 responses and T_H_17 cells differentiation (127, 128); and (iv) promotion of class-switch recombination from pro-inflammatory IgG1 to anti-inflammatory IgG4 antibodies (124, 129).

**Figure 2:**
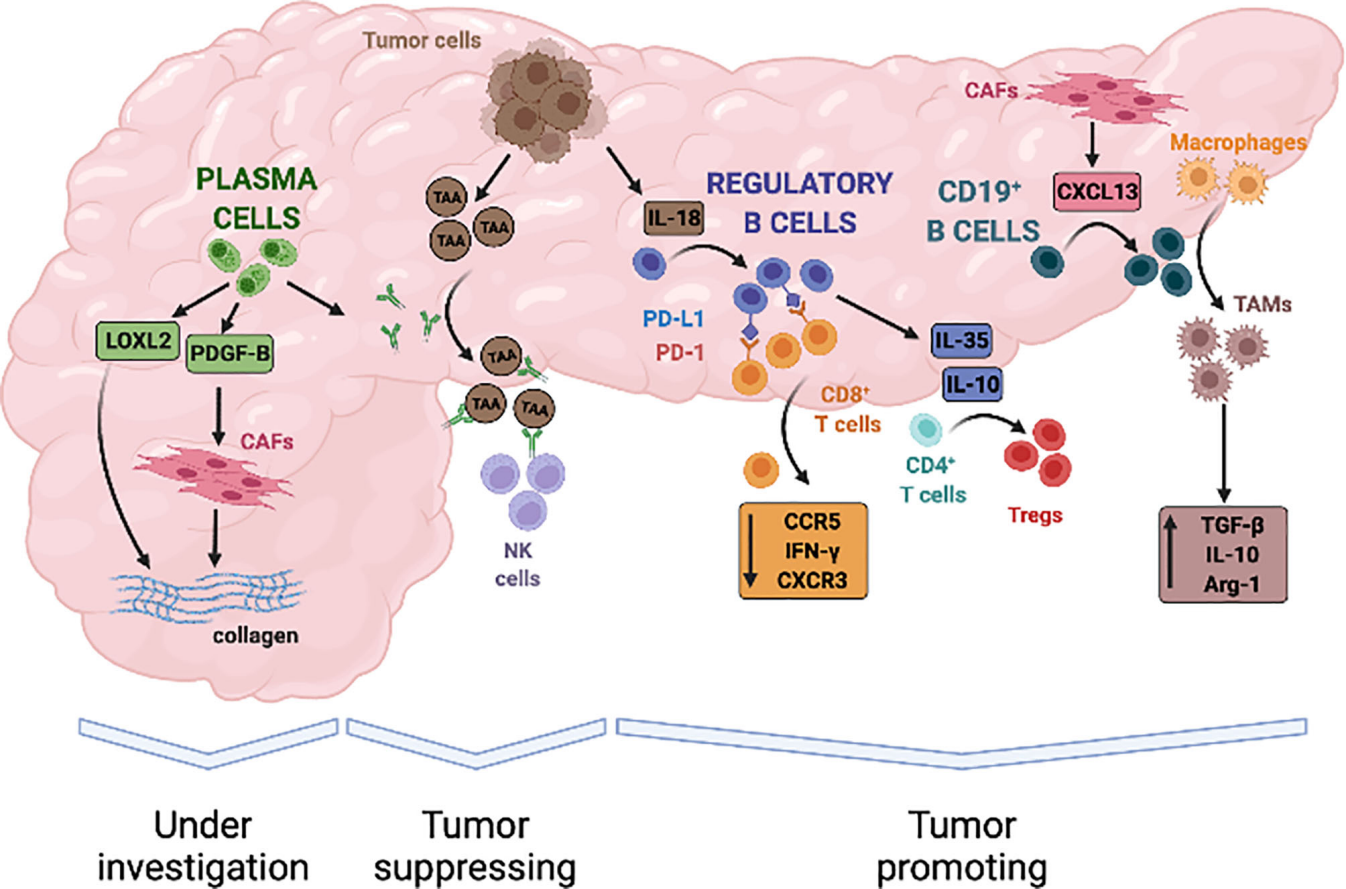
Evidence-based contribution of B lymphocytes to progression of pancreatic adenocarcinoma. B cells infiltrate PDAC in response to the release of local chemokines, such as CXCL13, and can contribute to both tumour promotion and tumour suppression through a variety of mechanisms. Tumour derived IL-18 can induce B lymphocytes with a regulatory phenotype that inhibit anti-tumour cytotoxic T cells responses. B-regulatory cells also secrete immune-modulatory cytokines such as IL-35 and IL-10 that induce T-regulatory cells (Tregs), stimulate tumour proliferation, and promote local angiogenesis. In addition, B-regulatory cells can recruit monocytes to tumour site and foster their differentiation into TGF-β and IL-10 producing M2 macrophages, thus further amplifying immune-evasive strategies and impairing cytotoxic responses. On the other hand, tumour-suppressive activity of B-cells in PDAC has not been clearly established but *in-vitro* studies suggest that it might depend on the production of autoantibodies against tumour associated antigen (TAA), engagement of natural killer cells (NK cells), and antigen dependent cell-mediated cytotoxicity (ADCC). Finally, plasma cells can sustain activation of the tumour stroma through the secretion of proliferative stimuli for cancer-associated fibroblasts (CAFs), such as PDGFB, and the production of enzymes that regulate extracellular matrix stiffness, such as LOXL2. Whether these properties ultimately exert tumour suppressive or tumour promoting effects is currently under investigation.

In PDAC, Breg cells are expanded compared to healthy individuals and their counts in the peripheral blood correlate with tumor stage and poor survival (130). Animal models suggest that Breg cells and PDAC cells engage in mutual activating interactions ultimately leading to tumor progression. In particular, pancreatic cancer cells reportedly induce Breg cells expansion by secreting IL-18 and Breg cells, in turn, support immune escape processes and tumor growth by expressing the programmed cell-death protein-1 ligand (PD-L1) and IL-35 (66, 130–132) (**Figure 2**).

PD-L1 is the natural ligand of the T-cell co-inhibitory receptor PD-1 expressed on antigen-presenting cells, and interaction of these two membrane molecules prevents full cytotoxic T-cell activation in physiological condition thus contributing to the removal of auto-reactive T lymphocytes and to the maintenance of immune tolerance (133). PD-L1 is overexpressed in several solid human cancers, including PDAC, and represents an established mechanisms exploited by neoplastic cells to promote tumor escape from immune-surveillance (134, 135). B-regulatory cells from patients with PDAC also express high levels of PD-L1 and *in vitro* interact with PD-1 expressing CD8^+^ T cells leading to reduced proliferation and IFN-γ secretion (131, 136) (**Figure 2**). Combined inhibition of IL-18 and PD-L1/PD-1 pathways with synthetic inhibitors reduces PDAC growth and metastasis formation in orthotopic animal models, reinforcing the *in vivo* relevance of the crosstalk between Breg and cancer cells for the progression of pancreatic cancer (130).

B regulatory cells - and specifically a subset of CD19^+^CD1d^hi^CD5^+^ Breg cells - can also influence tumor progression *via* secretion of IL-35 (66, 132). IL-35 is increased in the serum of PDAC patients compared to healthy individuals, and promotes immunosuppression and tumor growth in orthotopic murine models (137). By inducing the phosphorylation of STAT3, in fact, IL-35 causes a reduced expression of the chemotactic receptors CXCR3 and CCR5 as well as of interferon γ (IFN-γ) in CD8^+^ T cells (137). In addition, IL-35 suppresses endogenous antitumor T-cell responses *in vivo* by reducing CD4^+^ effector T cells and by increasing Treg cells in the tumor infiltrate (138). Breg cell derived IL-35 has been also shown to stimulate the proliferation of pancreatic cancer cell lines *in vitro* therefore harboring direct mitogenic properties (139) (**Figure 2**). Notably, PDAC growth in IL-35 deficient mice is significantly reduced and accompanied by a robust infiltration of activated cytotoxic CD8^+^ T cells, especially when combined with anti-PD-1 therapy (138). Conversely, growth deficiency of orthotopic pancreatic neoplasms in μMT mice can be rescued by the reconstitution of IL-35 producing CD1d^hi^CD5^+^ B-cells (66).

Finally, activation of the Bruton tyrosine kinase (BTK) in B-cells has been shown to foster reprogramming of tumor infiltrating macrophages towards a M2 phenotype thus impairing CD8^+^ T-cell anti-tumor responses and supporting PDAC growth (62, 65, 140) (**Figure 2**). In particular, BTK is activated in CD20^+^ B lymphocytes infiltrating PDAC compared to peripheral leukocytes and plays an important role in the differentiation of Breg cells and in the recruitment of myeloid cells to tumor site (141, 142). Interestingly, treatment of an orthotopically implanted KrasG12D-PanIN lesions with the BTK inhibitor tirabrutinib reduced the number of intra-tumoral CD1d^hi^CD5^+^ Breg cells while only moderately affecting total CD19^+^ B cells (140). This effect was associated with a decreased production of the immunosuppressive cytokines IL-10 and IL-35 at tissue site, with an increased IFNγ^+^CD8^+^ T cells recruitment and, ultimately, with a delayed progression to PDAC (140). Similarly, ibrutinib - another BTK inhibitor - combined with gemcitabine slowed tumor growth of orthotopic PDAC in a T-cell dependent manner (65). BTK inhibition, in fact, reprogrammed macrophages towards a M1 phenotype and restored CD8^+^ T cell cytotoxicity, thereby improving responsiveness to standard chemotherapy and inhibiting PDAC growth (65). Of note, efficacy of ibrutinib at limiting PDAC growth was previously associated with a reduced stromal reaction and with a decreased infiltrate of BTK expressing mast cells in both transgenic mice and patient-derived xenograft models (143).

Taken together this accumulating evidence suggest that B lymphocytes, specifically Breg cells, bear a spectrum of non-redundant tumor-promoting properties in both early and late PDAC stage, and that most of these properties are tightly linked to modulation of cytotoxic anti-tumor responses. A clinical trial investigating the relevance of targeting B-cells with the BTK inhibitor ibrutinib in metastatic PDAC has been recently completed and results are discussed in the last session of this review (144).

## Anti-Tumor Properties of B-Lymphocytes in Pancreatic Adenocarcinoma

As previously discussed most available murine studies point to a tumor-promoting role for B lymphocytes in PDAC (130, 138, 140). Yet, in analogy with other solid cancers, clinical data indicate that tumor-infiltrating B cells represent a positive prognostic factor also in pancreatic cancer, especially when organized in functional lymphoid structures, raising a controversy of crucial relevance for the implementation of effective immunotherapeutic strategies in this lethal tumor (75). In this regard, we are aware of a single study supporting anti-tumor properties of B cells in PDAC (60).

In this study B-cell activation and the effects of B-cell depletion were compared in an injectable orthotopic model of syngeneic KPC derived cell lines and in the KPC mouse, the murine model that most faithfully recapitulates PDAC pathology in terms of stromal reaction and immunological infiltrate. Notably, the authors observed a pronounced B cell infiltrate only in the latter whilst orthotopic tumors exhibited a relative paucity of B and T lymphocytes. B-cells in the KPC mouse also displayed markers of B cell activation (germinal center entry, B cell memory, and plasma cell differentiation) compared to orthotopic tumors and a significant intratumoral immunoglobulin deposition. Furthermore, in contrast to the accepted view of immunosuppressive properties of tumor infiltrating B cells, intratumoral B lymphocytes in both the KPC and orthotopic model up-regulated several pro-inflammatory genes and showed reduced expression of the immunomodulatory IL-35 and IL-10 genes. These results underscore the importance of using relevant preclinical tumor models when assessing B cell function as differences in B cell phenotype may be related to the genetic background of the animal, to the manipulation of orthotopic models, and to differential activation of circulating lymphocytes and of those infiltrating PDAC and secondary lymphoid organs. Indeed, while genetic deletion of B cells in μMT mice resulted in reduced orthotopic tumor growth this was not recapitulated by treatment of orthotopically injected immunocompetent mice with B-cell-depleting anti-CD20 antibody. Delayed tumor growth was also not observed in anti-CD20-treated KPC mice suggesting that B lymphocytes do not favor PDAC progression but rather participate to anti tumor responses as pointed by correlative human studies. Of note, most evidence supporting a tumor promoting role of B cells in PDAC has been obtained in orthotopically injected μMT mice, raising the possibility that reduced tumor progression in these animal models might be, at least in part, related to their genetic background rather than to the lack of B cells. Yet, precise mechanisms implicated in putative PDAC suppressive functions of B lymphocytes remain to be deciphered.

## Evidence from Clinical Trials

At the time of writing the RESOLVE study represents the single available clinical trial addressing the utility of targeting B-cells in PDAC. In this randomized, multicenter, double-blind, placebo-controlled, phase III study ibrutinib was added to nab-paclitaxel and gemcitabine as first-line treatment of patients with metastatic PDAC (144).

Ibrutinib is a first-inclass inhibitor of BTK approved for the treatment of various B-cell malignancies where it has been shown to potentiate antitumor activity of standard chemotherapy regimens by decreasing detrimental angiogenesis and desmoplasia as well as by increasing T-cell mediated cytotoxicity (145–148). The rationale for investigating ibrutinib plus nab-paclitaxel and gemcitabine stems from preclinical evidence whereby the combination of these drugs was shown to reduce tumor growth and to increase effector CD8^+^ T cells activity in an orthotopic model of PDAC (65). In addition, a phase Ib study of PDAC patients treated with ibrutinib before receiving gemcitabine and nabpaclitaxel showed that ibrutinib alone induces immunomodulatory changes in circulating and intramural lymphocytes obtained by endoscopic ultrasound-guided biopsies (149).

Based on these emerging research lines, the RESOLVE trial enrolled 424 patients with metastatic PDAC and randomized them to receive oral ibrutinib (560 mg once daily) (n = 211) or placebo (n = 213) in addition to standard therapy with nab-paclitaxel and gemcitabine. The primary endpoints were overall survival (OS) and investigator-assessed progression-free survival (PFS). With a hazard ratio (HR) of 1.109 (95% confidence interval (CI): 0.903-1.363), the OS of the experimental arm (median = 9.7 months) was not significantly different from the placebo arm (median = 10.8 months) (p value = 0.3225), whilst the PFS was significantly lower in patients treated with ibrutinib (median = 5.3 months) compared to those treated with placebo (median = 6.0 months) (HR = 1.564; 95% CI: 1.277-1.916; p value < 0.0001), suggesting an even potential worse trend.

Owing to the multiple rationales for targeting BTK pathways derived from animal models of PDAC, it is difficult to explain the reasons for the failure of ibrutinib in the RESOLVE trial. Bruton’s tyrosine kinase, in fact, has been implicated the differentiation of many tumor promoting cell lines including Breg cells, M2 macrophages, and mast cells, suggesting non redundant mechanisms equally concurring to immune escape and tumor progression (62, 65, 140, 143). On the one hand it is possible that not all BTK associated pathways identified in animal models are applicable to human disease or consistently inform on the role of B cells in PDAC progression. On the other hand, patient selection in the RESOLVE trial was not based on immunological parameters and we lack information about effective target engagement by ibrutinib, making it impossible to correlate disease outcomes with treatment related perturbations of the B-cell compartment.

Overall, the RESOLVE study does not fully clarify the utility of targeting B cells in pancreatic cancer, but this seminal experience should inform future clinical trials with immune-modulating agents in PDAC on a better patient phenotyping and on an improved study design in order to retrieve useful immunological data for optimizing precision medicine.

## Discussion

As current chemotherapeutic regimens - including checkpoint inhibitors - are only marginally effective in prolonging patient survival, there remains an unmet need for novel and innovative approaches for PDAC (150). In the era of personalized cancer medicine B-cells represent an increasingly attractive target for the complex pathophysiology of pancreatic cancer because most available animal models are concordant in pointing at a tumor-promoting role of this overlooked immune cell population. Yet, clinical-pathological evidence indicates that the presence of B-lymphocytes in pancreatic cancer correlate with a better prognosis raising a controversy of central relevance for the implementation of innovative immunotherapeutic strategies.

Looking ahead, as demonstrated by the ibrutinib experience, nonspecific B-cell depletion will very unlikely result in clinical benefit for all PDAC patients. A thorough understanding of the B-cell biology in PDAC will be therefore key to unveil potentially targetable tumor promoting and/or immunosuppressive interactions with neoplastic as well as with other immune and stromal cells. In addition, characterization of B-cell subsets with opposite roles in PDAC progression will instruct patient immunophenotyping and identification of subjects who will most likely benefit from targeted immune modulating therapies.

## Author Contributions

All authors contributed to the design of the work, acquisition, analysis and interpretation of data. All authors revised the work critically for important intellectual content and approved the final version of the manuscript. All authors agree to be accountable for all aspects of the work in ensuring that questions related to the accuracy or integrity of any part of the work are appropriately investigated and resolved. All authors contributed to the article and approved the submitted version.

## Funding

This work was supported by the Italian Association for Cancer Research (MFAG 2021 ID 26135 to ED-T), and by the Foundation for Research in Rheumatology (FOREUM Career Award 2021 to ED-T).

## Conflict of Interest

The authors declare that the research was conducted in the absence of any commercial or financial relationships that could be construed as a potential conflict of interest.

## Publisher’s Note

All claims expressed in this article are solely those of the authors and do not necessarily represent those of their affiliated organizations, or those of the publisher, the editors and the reviewers. Any product that may be evaluated in this article, or claim that may be made by its manufacturer, is not guaranteed or endorsed by the publisher.
